# Multimodal imaging characteristics and case management of intraspinal *Spirocerca lupi* infection in a dog

**DOI:** 10.1093/jvimsj/aalag286

**Published:** 2026-10-29

**Authors:** Alyssa J Carrillo, Federico R Vilaplana Grosso, Heather D S Walden, Oscar Alas, Gabriel A Garcia

**Affiliations:** Department of Small Animal Clinical Sciences, College of Veterinary Medicine, University of Florida, Gainesville, FL 32610, United States; Department of Small Animal Clinical Sciences, College of Veterinary Medicine, University of Florida, Gainesville, FL 32610, United States; Department of Small Animal Clinical Sciences, College of Veterinary Medicine, University of Florida, Gainesville, FL 32610, United States; Department of Small Animal Clinical Sciences, College of Veterinary Medicine, University of Florida, Gainesville, FL 32610, United States; Department of Small Animal Clinical Sciences, College of Veterinary Medicine, University of Florida, Gainesville, FL 32610, United States

**Keywords:** CT, MRI, parasitology, radiography, surgery, ultrasound

## Abstract

A 1-year-old female spayed mixed breed dog was ambulatory with paraparesis and proprioceptive ataxia, absent conscious proprioception in pelvic limbs, and spinal pain with a T3-L3 neurolocalization on presentation. Thoracic radiographs revealed caudal mediastinal mass, pneumothorax, and pleural effusion. Magnetic resonance imaging revealed multiple mediastinal masses with vertebral canal extension, extradural compression at T5-T6, intramedullary T2 hyperintensities at T5 and T9, esophageal compression and dilation, and aortic aneurysmal dilation. Transdiaphragmatic ultrasonography was suggestive of parasitic involvement. A right T5-T8 hemilaminectomy and parasitology confirmed *Spirocerca lupi*. Treatment with ivermectin (400 mcg/kg SC q24h for 3 days followed by once a week for 6 weeks), prednisone (0.7 mg/kg/day), and clindamycin (10.7 mg/kg PO q12h) was initiated. Recovery of independent ambulation occurred 2 months after treatment. Computed tomography revealed resolution of mediastinal and esophageal nodules with progressive aortic aneurysmal dilation. Multimodal imaging was essential for the management of spinal spirocercosis.

## Introduction

*Spirocerca lupi*, a parasitic nematode of dogs commonly found in tropical and subtropical regions, causes spirocercosis.^1^ Dogs, the definitive hosts, are infected by ingesting coprophagous beetles infected with third-stage larvae.^1^ The larvae are then released in the stomach, penetrate the gastric wall, migrate through the gastric and gastroepiploic arteries to the celiac artery, through the abdominal aortic wall to the thoracic aortic wall, and then through the thoracic aortic wall to the esophagus, where they typically form granulomatous nodules.^1–3^ Aberrant migrations are reported in the nervous system and are postulated to originate from the thoracic aortic wall through the intercostal arteries and their spinal branches to the extradural space and spinal cord parenchyma.^2–6^ An acute onset with rapidly progressive asymmetric paresis or paralysis accompanied by spinal pain in young dogs is described in the clinical manifestation of spinal spirocercosis.^6^ An important clinical feature of spinal spirocercosis is the changing neuroanatomic localization during the course of the disease.^6^ Various treatment protocols are described in the management of spinal spirocercosis.^6^^,^^7^ Multimodal imaging of spinal spirocercosis is minimally reported.^8–10^ CT imaging after treatment of spinal spirocercosis has not been described. The current case report describes multimodal imaging and case management of spinal spirocercosis in one dog.

## Case description

A 1-year-old 6.9-kg female spayed mixed breed dog was presented to the University of Florida Small Animal Teaching Hospital (UFSATH) for evaluation of spinal pain, paraparesis, and inappetence. The dog was originally from Nicaragua but had resided in Florida for approximately 4 months before presentation. One month before presentation, the dog had a positive *Dirofilaria immitis* antigen test, where treatment with prednisone at a tapering dose (0.63 mg/kg PO q12h for 7 days, 0.63 mg/kg PO q24h for 7 days, 0.63 mg/kg PO every other day for 7 days), doxycycline (9.5 mg/kg PO q12h for 28 days), and ivermectin/pyrantel (Heartgard, Boehringer Ingelheim; 8.6 mcg/kg/7.2 mg/kg PO once) was initiated. Two weeks before presentation, the dog was evaluated for unspecified pain suspected to be secondary to a cracked molar and was administered carprofen (1.6 mg/kg PO q12h). Additionally, the dog experienced episodes of suspected regurgitation at this time. Two days before presentation, the dog was noted to flail, urinate, and vocalize. A similar event occurred the next day.

Upon presentation to UFSATH, the dog was noted to be ambulatory with moderate paraparesis and proprioceptive ataxia. Conscious proprioception was absent in the pelvic limbs, and spinal pain was appreciated at the thoracolumbar junction. Lesions were neurolocalized to T3-L3. Complete blood count revealed a mild normocytic, normochromic, non-regenerative anemia (hematocrit 34%, normal, 37.3%-61.7%; mean corpuscular volume 71.5 fL, normal, 61.6-73.5 fL; mean corpuscular hemoglobin concentration 35.6 g/dL, normal, 32-37.9 g/dL), moderate leukocytosis (21.27 K/μL, normal, 4.6-11.6 K/μL) characterized by neutrophilia (17.05 K/μL, normal, 2.6-8 K/μL) and monocytosis (1.07 K/μL, normal, 0.1-0.9 K/μL), and moderate thrombocytosis (541 K/μL, normal, 130-328 K/μL). Blood chemistry revealed mild hyperproteinemia (9.8 g/dL, normal, 5.2-8.2 g/dL) driven by hyperglobulinemia (6.9 g/dL, normal, 2.5-4.5 g/dL). A 4DX SNAP test (IDEXX, Westbrook, ME) was *D. immitis* antigen positive. A Modified Knott’s test revealed microfilariae present with morphology suggestive of *Acanthocheilonema reconditum* and confirmed via polymerase chain reaction (PCR). PCR was also negative for *D. immitis*. Thoracic radiographs revealed a large, caudal mediastinal mass likely esophageal in origin, scant pneumothorax, mild pleural effusion, and visceral pleural thickening compatible with pleuritis (Figure 1).

**Figure 1:**
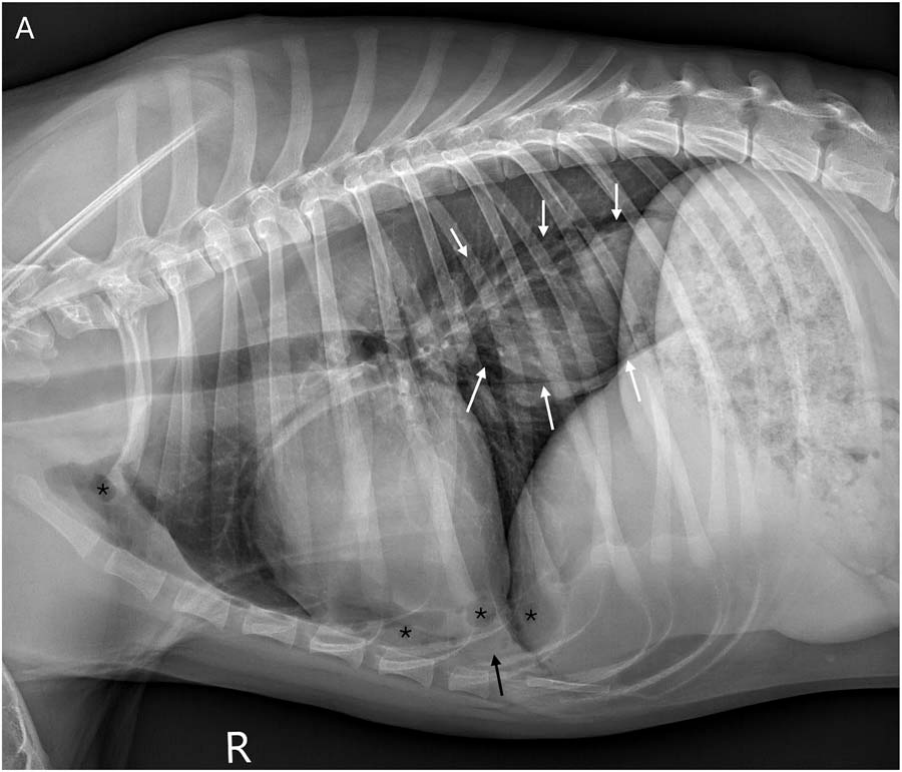
Right lateral projection of the thorax showing a caudal dorsal mediastinal mass (arrows) as well as a scant pneumothorax (asterisks) and pleural effusion (black arrow). The mass is suspected to be esophageal in origin. Along the craniodorsal aspect of the thorax, a thick, well-defined soft tissue opaque line is seen along the pulmonary margins, compatible with pleural thickening (ie, pleuritis).

The next day, the dog’s signs of neurologic disease progressed to paresis in the left pelvic limb and plegia in the right pelvic limb with intact sensation to pain. Lesions were neurolocalized to T3-L3. A T3-caudal MRI revealed a moderate amount of ill-defined, irregularly marginated T2W hyper and isointense, T1W isointense to the spinal musculature contrast-enhancing mass. Within the mass, there were multiple, well-defined, tubular T2W hyperintense and peripherally contrast-enhancing regions identified. The mass extends dorsally through the right T5-T6 intervertebral foramen into the right ventrolateral aspect of the vertebral canal, occupying 25% of the vertebral canal, extending cranially until the mid-aspect of C5 and caudally until the caudal aspect of C7 displacing the spinal cord. The spinal cord from the mid-aspect of T11 and cranially beyond the field of view was diffusely, mildly thickened, causing mild regional circumferential attenuation of the cerebrospinal fluid and epidural fat. The meninges were diffusely, mildly thickened and contrast enhancing at this level. In addition, a large partially obstructive caudal mediastinal mass likely arising from the esophagus with moderate diffuse orad thoracic esophageal dilation was noted. Other findings included mild aortic arch aneurysmal dilation and suspected aortic wall mineralization, scant pleural effusion and pneumothorax, mild middle tracheobronchial lymphadenopathy, and moderate generalized splenomegaly (Figure 2). For further assessment and potential surgical planning, a CT of the neck, thorax, and abdomen was performed revealing regionally extensive dorsal mediastinal and esophageal coalescing nodules and masses with extension into the vertebral canal and secondary extradural spinal cord compression, as well as secondary esophageal compression and segmental dilation suggestive of partial esophageal obstruction. Also, there was mild to moderate aortic arch and proximal aortic aneurysmal dilation, multifocal aortic wall mineralization, mild bilateral pneumothorax, multifocal peripheral areas of lung consolidation, scant pneumomediastinum, and scant pleural effusion. There was moderate cranial mediastinal and mild mandibular, medial retropharyngeal, tracheobronchial, and jejunal lymphadenomegaly, and moderate generalized splenomegaly (Figure 3). Immediately after the CT and while the dog was still anesthetized, a focal transdiaphragmatic ultrasonography of the suspected esophageal mass was performed showing multiple long, cylindrical structures suggestive of parasitic involvement (Figure 4). At the same time, ultrasonography-guided fine needle aspirates of the spleen and mediastinal mass were performed revealing extramedullary hematopoiesis and mesenchymal proliferation with mild to moderate cytologic atypia and mild lymphocytic infiltrate, respectively (Figure S1). An atlantooccipital cerebrospinal fluid tap revealed a clinically significant marked mixed neutrophilic and mononuclear pleocytosis (white blood cell 2600 K/μL; red blood cell 12 650 K/μL; Protein 280.7 mg/dL).

**Figure 2:**
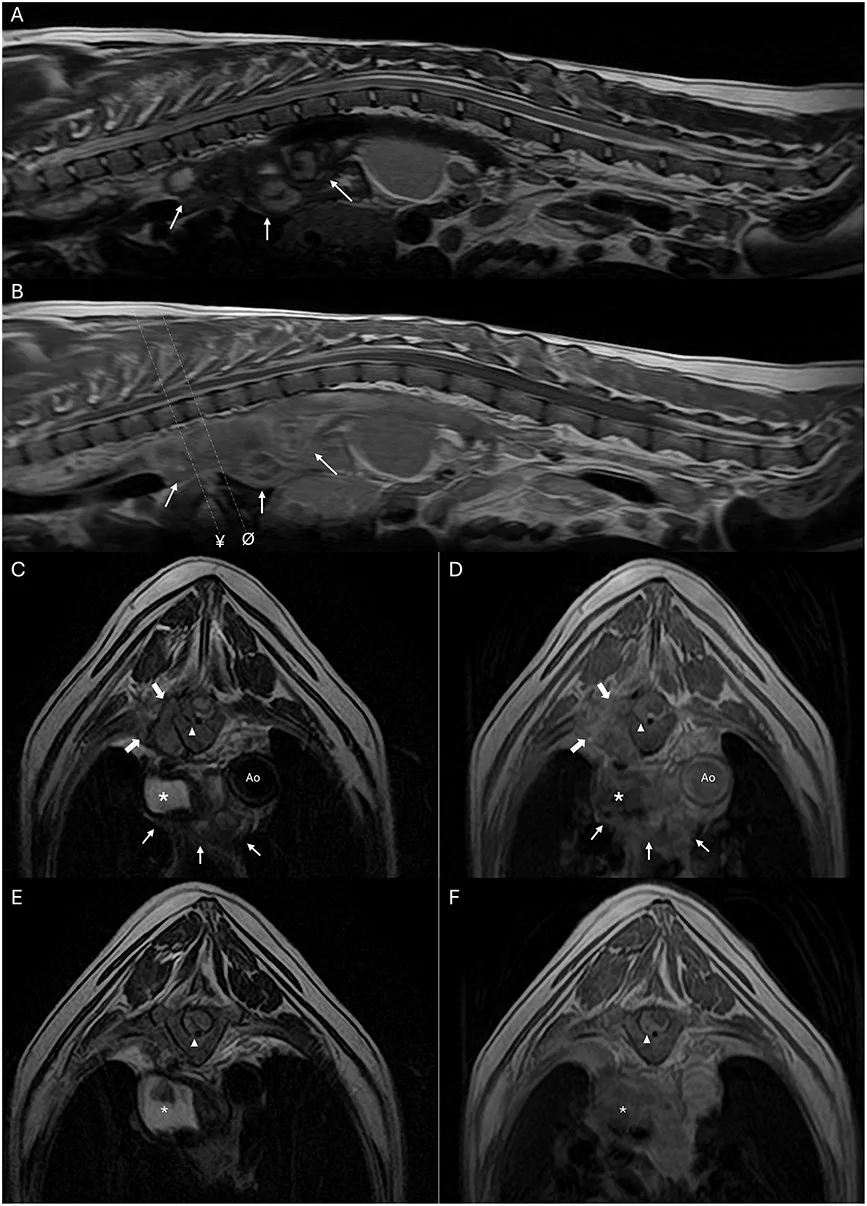
Sagittal T2-weighted (A) and T1-weighted postcontrast (B), as well as transverse T2-weighted (C, E) and T1-weighted postcontrast (D, F) images of the thoracic and lumbar spine. The dotted line ¥ indicates transverse images (C, D), and the dotted line Ø indicates transverse images (E, F). In the caudodorsal mediastinum, at the level of T9-T11, there are multiple, variably sized and variably shaped, well-defined masses and nodules that are peripherally T2W hypointense, centrally hyperintense, with strong peripheral contrast enhancement and centrally non-contrast enhancing (white arrows). The mediastinal masses cause extraluminal narrowing of the esophagus that results in moderate, diffuse orad esophageal dilation with a moderate amount of T2W hyperintense, T1W hypointense fluid (white asterisks). Extending along the craniodorsal mediastinum, involving the longus colli muscles bilaterally, there is a moderate amount of ill-defined, irregularly marginated, heterogeneously T2W hyper- to isointense, heterogeneously contrast-enhancing tissue (white wide arrows) which extends dorsally, through the right T5-T6 intervertebral foramen into the right ventrolateral aspect of the vertebral canal, occupying approximately 25% of the vertebral canal (white arrow heads). The descending aorta is mildly focally distended (Ao).

**Figure 3:**
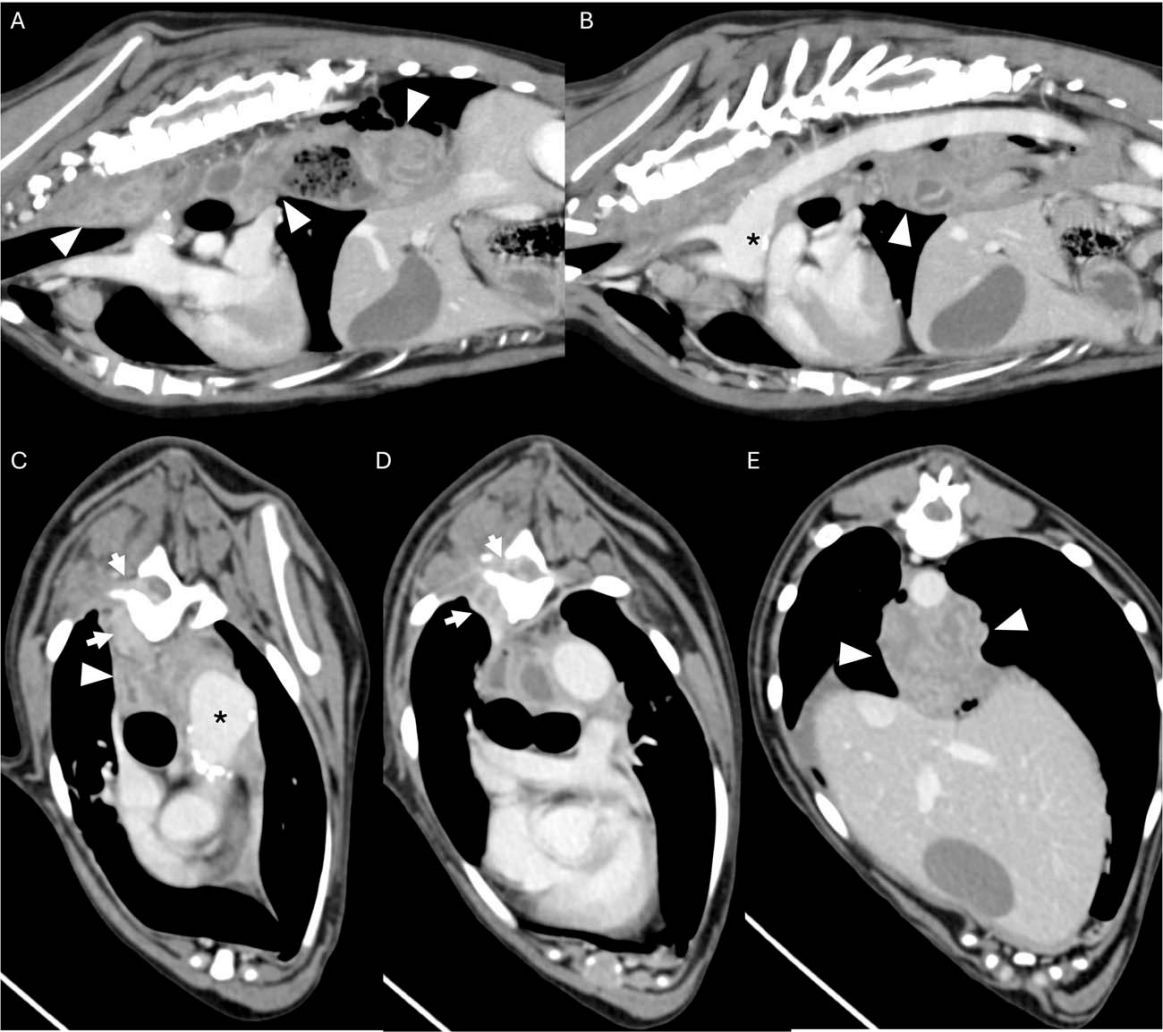
Sagittal postcontrast (A, B) and transverse postcontrast (C, D, E) CT images of the thorax in a soft tissue algorithm. Diffusely throughout the dorsal aspect of the mediastinum and surrounding the esophagus, there are innumerable, coalescing, rounded/lobular, heterogeneously soft tissue attenuating, primarily peripherally contrast-enhancing nodules and masses with central fluid attenuation (white arrow heads) as well as contrast-enhancing tissue. At the level of T4 through T6, there is heterogeneously contrast-enhancing tissue which extends toward the right-sided T5 and T6 intervertebral foramina and into the vertebral canal, resulting in moderate leftward displacement and deformation of the spinal cord. An aortic aneurysm is visible at the level of the aortic arch (asterisk) as well as multifocal wall mineralization. CT images were obtained with a slice thickness of 2 mm and are displayed with a window width of 400 and a window level of 40.

**Figure 4:**
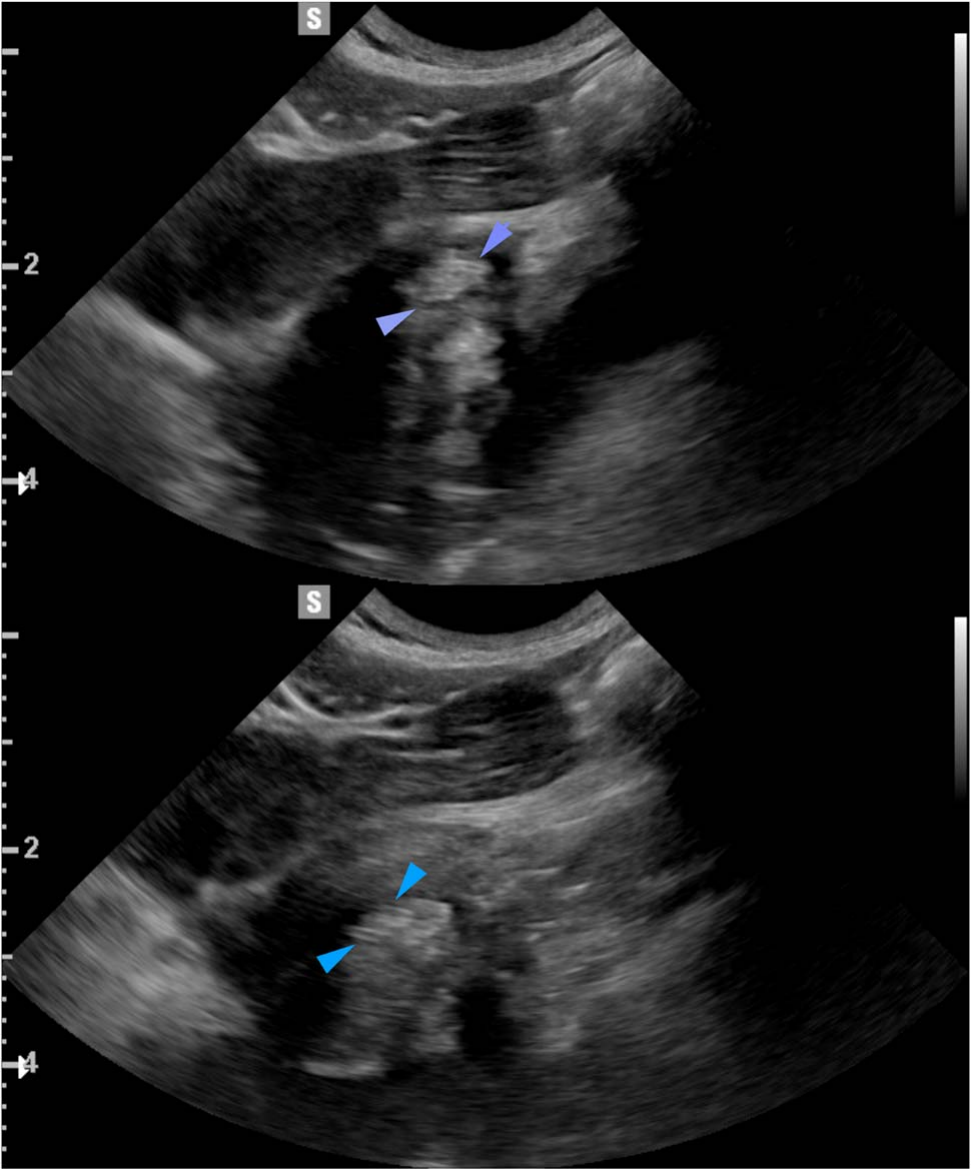
Ultrasonographic images of the caudal dorsal mediastinal mass through trans diaphragmatic approach. Within the mass, there are multiple thin, double walled, smoothly marginated structures resembling worms (blue arrowheads).

The next day, the signs of neurologic disease progressed and the dog developed a left head tilt, left Horner’s syndrome, paraplegia, paresis in the left thoracic limb, and delayed conscious proprioception in the left thoracic limb. Lesions were neurolocalized as multifocal, involving the left vestibular system, cervical spine, and T3-L3. Due to progressive neurological signs, a right T5-T8 hemilaminectomy with granuloma debulking was performed. Upon manipulation of the granuloma, 5 approximately 4 cm long red worms were identified and removed from the spinal canal (Video S1). The granuloma was removed in pieces until no additional material was identified. Parasite identification revealed adult male and female nematodes consistent with *S. lupi* (Figure S2). Histopathology of the granuloma revealed marked necrotizing and granulocytic fasciitis associated with frequent ova and rare larval nematodes. Aerobic and anaerobic tissue culture from the surgical site was negative for growth. Fecal sedimentation revealed spirurid ova consistent with *S. lupi* (Figure S2). Treatment with ivermectin (400 μg/kg SC) for 3 consecutive days followed by once a week for 6 weeks (9 total injections), clindamycin (10.7 mg/kg PO q12h) for the duration of ivermectin injections, and prednisone (0.7 mg/kg PO q24h) for the duration of ivermectin injections was initiated. Diphenhydramine (2 mg/kg IM) was administered before ivermectin injections. Physiotherapy was prescribed.

Throughout the course of injections, signs of neurologic disease waxed and waned. After completion of the final injection, the dog was nonambulatory tetraparetic with absent conscious proprioception in the pelvic limbs. Lesions were neurolocalized as multifocal, including the cervical spine and T3-L3. Prednisone was gradually tapered over a 6-week period. The dog recovered independent ambulation approximately 2 months after the initiation of treatment. The dog was presented to UFSATH 3 months after initiation of therapy. On neurological examination, the dog was ambulatory with mild to moderate paraparesis and pelvic limb proprioceptive ataxia. A follow-up CT of the neck and thorax was performed 2 months after completion of injections and revealed resolution of the previously noted multifocal mediastinal masses and nodules, multifocal peripheral pulmonary consolidation, pneumothorax, pneumomediastinum, pleural effusion, and tracheobronchial lymphadenopathy. There was mildly progressive aortic aneurysmal dilation and persistent aortic wall mineralization (Figure 5). Nine months after CT, continued improvement was noted by the owner via a verbal update.

**Figure 5:**
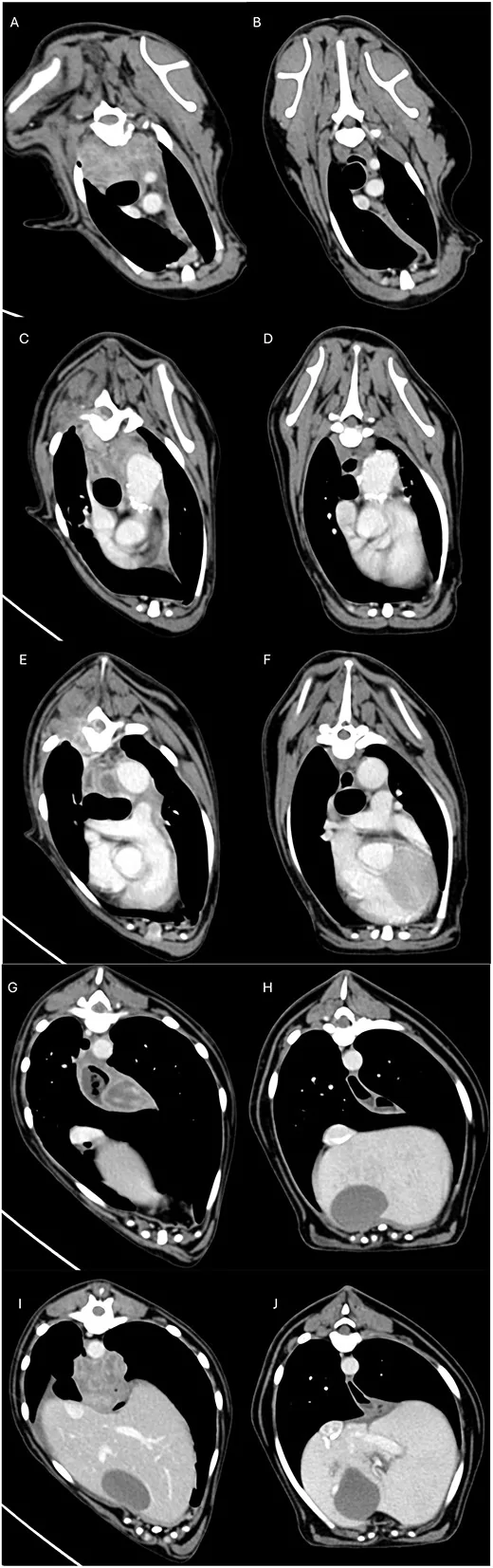
Transverse postcontrast CT images of the thorax in a soft tissue algorithm before (A, C, E, G, I) and 3 months after treatment (B, D, F, H, J) showing resolution of the previously noted multifocal mediastinal nodules and masses, as well as the vertebral canal extension and compressive myelopathy at T4-T6 (C-D, E-F). CT images were obtained with a slice thickness of 2 mm and are displayed with a window width of 400 and a window level of 40.

## Discussion

This report illustrates the multimodal imaging features and clinical management of spinal spirocercosis in a dog, highlighting the critical role of imaging in both diagnosis and treatment. Spinal involvement in *S. lupi* infection is a rare but well-documented manifestation of aberrant migration.^4–6^ The clinical presentation of spinal spirocercosis is described as a rapidly progressive, asymmetric paresis or paralysis accompanied by spinal pain.^1^^,^^2^^,^^6^ In the current case report, the dog exhibited a rapidly progressive, asymmetric deterioration in neurological status, consistent with previous studies. However, involvement of all 4 limbs in the disease is rarely reported in the veterinary literature.^6^ The variations in neuroanatomic localization observed in this case were noted in previous studies and are suspected to be due to spinal cord damage secondary to parasite migration through the parenchyma.^6^ Additionally, the dog in the current case report developed vestibular signs and Horner’s syndrome. The development of vestibular signs is speculated to be secondary to either a thromboembolic event or secondary to parasite migration while the development of Horner’s syndrome is speculated to be secondary to thoracic damage.

The dog had a travel history from Nicaragua. Although *S. lupi* has a global distribution, it is most prevalent in tropical and subtropical climates.^1^ As the dog was located in Florida at the time of clinical sign development, diagnosis before presentation was likely delayed given the location. In the current case report, the dog tested positive for *D. immitis* antigen on a 4DX SNAP test (IDEXX, Westbrook, ME) but negative on PCR testing, indicating a false positive. Serologic cross-reactivity has previously been documented between *S. lupi* and *D. immitis* antigens, potentially complicating the diagnosis of *S. lupi* in endemic areas.^11^

Thoracic vertebral spondylitis is a unique lesion associated with *S. lupi* infection.^1^^,^^3^^,^^5^^,^^10^^,^^12^ Vertebral spondylitis was not appreciated on any imaging modality in the current case report. Aortic aneurysmal dilation and mineralization are well-documented features of *S. lupi* migration, consistent with the current case report.^1^ Aortic aneurysmal dilation or thoracic vertebral spondylitis in conjunction with a caudodorsal mediastinal mass is considered pathognomonic for *S. lupi* infection radiographically.^13^^,^^14^ MRI revealed evidence of extradural spinal cord compression and T2-weighted intramedullary hyperintensities in the current case report, as previously observed.^2^^,^^9^ A limitation of the current report is the lack of a brain MRI despite the presence of intracranial signs. CT documented the extent of esophageal, spinal, and aortic pathology in the current case report and is described as a useful modality for evaluating *S. lupi* infection.^14^ Use of ultrasonography revealed evidence of suspected parasites in the esophageal nodule in the current case. Ultrasonography is used to document aortic wall abnormalities with poor sensitivity in dogs infected with *S. lupi*.^15^ Additionally, treatment after CT is not documented in the management of spinal spirocercosis to the authors’ knowledge. The current case report demonstrated resolution of the mediastinal and esophageal masses and nodules as well as associated pleural effusion, pulmonary peripheral consolidation, pneumothorax, and tracheobronchial lymphadenopathy. However, aortic aneurysmal dilation was mildly progressive, which suggests that structural changes associated with the vasculature might persist long-term despite appropriate management of parasitic burden. Endoscopy is the gold standard for diagnosis and management of esophageal spirocercosis.^2^ Endoscopy was not performed in the current case report; however, it should have been performed given the presence of esophageal nodules and potential for neoplastic transformation.

Treatment in this case report was based on previous studies with some modifications.^6^^,^^7^ Doramectin, a derivative of ivermectin, was not available at this institution; therefore, ivermectin was administered. In the previously described protocol, prednisone dosage was tapered every 3 days for 10 days.^7^ Inflammatory responses secondary to parasite movement and death are expected; therefore, anti-inflammatory steroids are indicated.^7^ In the current case, prednisone was administered for the duration of ivermectin administration and gradually tapered after cessation of ivermectin administration. Surgery is used in the treatment of spinal spirocercosis.^5^^,^^16^ Diphenhydramine was administered before ivermectin administration in the current case report to potentially mitigate hypersensitivity reactions secondary to parasite disruption, as described in the management of neurocuterebriasis.^17^ Based on a previous study, the recovery period can be prolonged, with a majority of dogs regaining ambulation within 3 months; however, involvement of all limbs is rarely reported.^6^ The dog regained independent ambulation approximately 2 months after the initiation of therapy. In conclusion, this case highlights the importance of multimodal imaging in diagnosing and managing spinal spirocercosis.

## Supplementary Material

Supplementary_Materials_aalag286

## Off-label antimicrobial declaration

The authors declare no off-label use of antimicrobials.

## Institutional animal care and use committee or other approval declaration

The authors declare no institutional animal care and use committee or other approval was needed.

## References

[1] Rojas A, Dvir E, Baneth G. Insights on *Spirocerca lupi*, the carcinogenic dog nematode. Trends Parasitol. 2020;36:52–63. 10.1016/j.pt.2019.10.004 31734099

[2] Chai O, Yas E, Brenner O, et al. Clinical characteristics of *Spirocerca lupi* migration in the spinal cord. Vet Parasitol. 2018;253:16–21. 10.1016/j.vetpar.2018.02.025 29604997

[3] Mazaki-Tovi M, Baneth G, Aroch I, et al. Canine spirocercosis: clinical, diagnostic, pathologic, and epidemiologic characteristics. Vet Parasitol. 2002;107:235–250. 10.1016/S0304-4017(02)00118-8 12127253

[4] Dvir E, Perl S, Loeb E, et al. Spinal intramedullary aberrant *Spirocerca lupi* migration in 3 dogs. J Vet Intern Med. 2007;21:860–864. 10.1111/j.1939-1676.2007.tb03033.x 17708411

[5] Du Plessis CJ, Keller N, Millward IR. Aberrant extradural spinal migration of *Spirocerca lupi*: four dogs. J Small Anim Pract. 2007;48:275–278. 10.1111/j.1748-5827.2006.00262.x 17425698

[6] Casoy YL, Srugo I, Barnoon I, et al. Aberrant migration of *Spirocerca lupi* to the spinal cord in dogs: association of clinical signs, CSF analysis, preventive treatment, and outcome. Vet J. 2025;309:106286. 10.1016/j.tvjl.2024.106286 39667609

[7] Asiag N, Chai O, Yodovner S, et al. Evaluation of a treatment protocol in dogs with intraspinal spirocercosis. J Am Vet Med Assoc. 2023;261:384–390. 10.2460/javma.22.09.0401 36476412

[8] Lindsay N, Kirberger R, Williams M. Imaging diagnosis—spinal cord chondrosarcoma associated with spirocercosis in a dog. Vet Radiol Ultrasound. 2010;51:614–616. 10.1111/j.1740-8261.2010.01718.x 21158232

[9] Chai O, Shelef I, Brenner O, Dogadkin O, Aroch I, Shamir MH. Magnetic resonance imaging findings of spinal intramedullary spirocercosis. Vet Radiol Ultrasound. 2008;49: 456–459. 10.1111/j.1740-8261.2008.00407.x 18833954

[10] Kirberger RM, Clift SJ, van Wilpe E, Dvir E. *Spirocerca lupi*-associated vertebral changes: a radiologic-pathologic study. Vet Parasitol. 2013;195:87–94. 10.1016/j.vetpar.2012.12.039 23298568

[11] Aroch I, Rojas A, Slon P, Lavy E, Segev G, Baneth G. Serological cross-reactivity of three commercial in-house immunoassays for detection of *Dirofilaria immitis* antigens with *Spirocerca lupi* in dogs with benign esophageal spirocercosis. Vet Parasitol. 2015;211:303–305. 10.1016/j.vetpar.2015.06.010 26116456

[12] Giannelli A, Baldassarre V, Ramos RAN, et al. *Spirocerca lupi* infection in a dog from southern Italy: an “old fashioned” disease? Parasitol Res. 2014;113:2391–2394. 10.1007/s00436-014-3912-y 24781025

[13] Thong P, Hopper B, Tenni G, Lenard Z. Thoracic radiographic findings of canine spirocercosis in Australia. Aust Vet J. 2025;103:354–367. 10.1111/avj.13447 40312276

[14] Dvir E, Kirberger RM, Malleczek D. Radiographic and computed tomographic changes and clinical presentation of spirocercosis in the dog. Vet Radiol Ultrasound. 2001;42:119–129. 10.1111/j.1740-8261.2001.tb00914.x 11327359

[15] Merhavi N, Segev G, Dvir E, Peery D. Ultrasonography is insensitive but specific for detecting aortic wall abnormalities in dogs infected with *Spirocerca lupi*. Vet Rec. 2020;187:e59–e59. 10.1136/vr.105836 32759378

[16] Klainbart S, Chai O, Vaturi R, Rapoport K, Aroch I, Shamir MH. Nematode eggs observed in cytology of cerebrospinal fluid diagnostic for intramedullary *Spirocerca lupi* spinal cord migration. Vet Clin Pathol. 2018;47:138–141. 10.1111/vcp.12560 29329490

[17] Chen XYW, James FMK. Antemortem diagnostic tests and treatment outcomes using ivermectin in 13 cases of presumptive feline neurocuterebriasis. Can Vet J. 2025;66:64–69. 10.1002/jvmi.202400030 PMC11705184 39781413

