# Supplementary material for: Multimodal imaging characteristics and case management of intraspinal Spirocerca lupi infection in a dog
Source: J Vet Intern Med. 2026 Oct 29;40(5):aalag286. doi: 10.1093/jvimsj/aalag286 (PMC13718424; doi:10.1093/jvimsj/aalag286)
Supplement: Supplementary file 1 [file supplementary_materials_aalag286.zip › Supplementary_Materials_aalag286/Supplemental_caption_aalag286.docx]

Supplemental Video 1: Removal of *Spirocerca lupi* worm from spinal canal through right T5-T8 hemilaminectomy after granuloma debulking.

Supplemental Figure 1: Photomicrograph of mediastinal mass fine needle aspirate. Mesenchymal cell (arrow) is oval to stellate with variably distinct borders and moderate amounts of light to deep blue cytoplasm that contains small vacuoles. Moderately blood contaminated background.

Supplemental Figure 2: Photomicrograph of *Spirocerca lupi* anterior (A), male *Spirocerca lupi* posterior spicules (B), and fecal sedimentation of *Spirocerca lupi* ova (arrow) measuring 36.5 µm at 400x total magnification.
